# Probing the Acceptor Active Site Organization of the Human Recombinant β1,4-Galactosyltransferase 7 and Design of Xyloside-based Inhibitors*

**DOI:** 10.1074/jbc.M114.628123

**Published:** 2015-01-08

**Authors:** Mineem Saliba, Nick Ramalanjaona, Sandrine Gulberti, Isabelle Bertin-Jung, Aline Thomas, Samir Dahbi, Chrystel Lopin-Bon, Jean-Claude Jacquinet, Christelle Breton, Mohamed Ouzzine, Sylvie Fournel-Gigleux

**Affiliations:** From the ‡ UMR 7365 CNRS-Université de Lorraine, Biopôle-Faculté de Médecine, CS 50184, 54505 Vandoeuvre-lès-Nancy Cedex,; the § University Grenoble Alpes, CERMAV, BP 53, 38041 Grenoble Cedex 9, and; the ¶ UMR 7311 CNRS-Institut de Chimie Organique et Analytique, Université d'Orléans-Pôle de Chimie, Rue de Chartres, 45067 Orléans Cedex 02, France

**Keywords:** Enzyme Inhibitor, Enzyme Kinetics, Glycosaminoglycan, Glycosyltransferase, Proteoglycan Synthesis, Site-directed Mutagenesis

## Abstract

Among glycosaminoglycan (GAG) biosynthetic enzymes, the human β1,4-galactosyltransferase 7 (hβ4GalT7) is characterized by its unique capacity to take over xyloside derivatives linked to a hydrophobic aglycone as substrates and/or inhibitors. This glycosyltransferase is thus a prime target for the development of regulators of GAG synthesis in therapeutics. Here, we report the structure-guided design of hβ4GalT7 inhibitors. By combining molecular modeling, *in vitro* mutagenesis, and kinetic measurements, and *in cellulo* analysis of GAG anabolism and decorin glycosylation, we mapped the organization of the acceptor binding pocket, in complex with 4-methylumbelliferone-xylopyranoside as prototype substrate. We show that its organization is governed, on one side, by three tyrosine residues, Tyr^194^, Tyr^196^, and Tyr^199^, which create a hydrophobic environment and provide stacking interactions with both xylopyranoside and aglycone rings. On the opposite side, a hydrogen-bond network is established between the charged amino acids Asp^228^, Asp^229^, and Arg^226^, and the hydroxyl groups of xylose. We identified two key structural features, *i.e.* the strategic position of Tyr^194^ forming stacking interactions with the aglycone, and the hydrogen bond between the His^195^ nitrogen backbone and the carbonyl group of the coumarinyl molecule to develop a tight binder of hβ4GalT7. This led to the synthesis of 4-deoxy-4-fluoroxylose linked to 4-methylumbelliferone that inhibited hβ4GalT7 activity *in vitro* with a *K_i_* 10 times lower than the *K_m_* value and efficiently impaired GAG synthesis in a cell assay. This study provides a valuable probe for the investigation of GAG biology and opens avenues toward the development of bioactive compounds to correct GAG synthesis disorders implicated in different types of malignancies.

## Teaser

**Background:** Glycosyltransferase inhibitors have important applications in therapeutics and as chemical biology tools.

**Results:** The human β1,4-galactosyltransferase 7 enzyme active site was mapped by modeling, mutagenesis, and *in vitro*/*in cellulo* assays, and novel inhibitors were synthesized.

**Conclusion:** An efficient inhibitor of β1,4-galactosyltransferase 7 and glycosaminoglycan synthesis was obtained.

**Significance:** This inhibitory molecule can be exploited to investigate glycosaminoglycan biology and modulate glycosaminoglycan synthesis in therapeutics.

## Introduction

Glycosaminoglycans (GAGs)^2^ are linear heteropolysaccharide chains covalently attached to the core protein of a variety of proteoglycans (PGs). Because of their high structural diversity and their anionic characteristics, GAGs interact with a network of cellular and extracellular mediators including cytokines and chemokines, enzymes and enzyme inhibitors, matrix proteins, and membrane receptors (1). There is currently great emphasis on the crucial roles of GAGs in numerous physiological events including cell differentiation, proliferation and migration (2), and its pathological aspects, such as tumor formation, progression, and metastasis (3). Furthermore, because PGs are ubiquitously expressed in extracellular matrices and on cell surfaces of virtually every tissue, they are also involved in the normal and pathological functions of the cardiovascular and osteoarticular system (4), in amyloid disorders (5) and in axonal de- and regeneration (6). GAG biosynthesis is initiated by the formation of a tetrasaccharide linkage region (GlcAβ1–3Galβ1–3Galβ1–4Xylβ1-O-) covalently linked to serine residues of the PG core protein (7). This tetrasaccharide acts as a primer for the elongation of major GAG chains, *i.e.* chondroitin/dermatan sulfate or heparin/heparan sulfate, which polymerization involves the coordinated activities of chondroitin-sulfate synthases and heparan-sulfate synthases (exostosins, EXT), respectively (8, 9). Mature GAG chains are finally produced by the modifications of their constitutive disaccharide units catalyzed by epimerases and sulfotransferases, which considerably increase their structural and functional diversity (10, 11).

The human xylosylprotein β1,4-galactosyltransferase (EC 2.4.1.1337, hβ4GalT7) catalyzes the transfer of the first Gal residue of the tetrasaccharide linkage from the activated sugar UDP-galactose (UDP-Gal) onto Xyl residues attached to the PG core protein (12). Because all GAGs share the same stem core tetrasaccharide, β4GalT7 is a central enzyme in GAG biosynthesis. Indeed, hβ4GalT7 mutations have been associated with a rare genetic condition, the progeroid form of Ehlers-Danlos syndrome (EDS), a group of connective tissue disorders characterized by a major deficiency in PG synthesis. As a consequence of GAG defect, EDS patients exhibit motor development delay, and musculoskeletal malformations, hypermobile joints, and wound healing defaults (13). Patients gene sequencing revealed the presence of missense mutations leading to L206P, A186D (14, 15), and R270C substitutions (16) in the catalytic domain, resulting in a partially or totally inactive enzyme. Recently, we showed that R270C replacement reduced affinity toward the xyloside acceptor and strongly affected GAG chains formation in β4GalT7-deficient CHOpgsB-618 cells (17). There is currently no effective therapy for treating EDS patients.

Interestingly, the biosynthesis of GAGs can be manipulated by simple xylosides carrying a hydrophobic aglycone, which act as substrates and/or inhibitors of hβ4GalT7. Xyloside analogs have been shown to efficiently induce GAG synthesis bypassing the natural Xyl-substituted core protein of PGs for several decades (18, 19). The xyloside-primed GAG chains are usually excreted and show interesting biological functions such as activation of fibroblast growth factor (FGF) signaling (20, 21), antithrombotic (22), tissue regenerating (23), anti-angiogenic (24) and anti-proliferative properties (25, 26). In addition, several groups have synthesized a series of xyloside analogs as potential inhibitors of GAG synthesis. Such compounds would represent highly valuable chemical biology tools to probe the functions of GAGs in cell systems and model organisms and as a starting point toward the development of pharmaceuticals, in particular anti-tumor agents. Recently, Garud *et al.* (27) and Tsuzuki *et al.* (28) used click chemistry to generate libraries of 4-deoxy-4-fluorotriazole analogs comprising a set of hydrophobic molecules appended to the anomeric carbon of the xyloside. Siegbahn *et al.* (29, 30) developed a collection of naphthyl and benzyl xylosides substituted on different positions of the Xyl moiety. These studies led to the discovery of promising xyloside-derived inhibitors of GAG synthesis when screened in cell models.

However, until recently, the development of substrates and inhibitors of β4GalT7 has been mostly limited to the synthesis of libraries of analog compounds and their testing in cell assays. Toward the rational design of hβ4GalT7 inhibitors, we have been involved in structure-activity relationship studies of the recombinant human enzyme for several years and identified critical active site amino acids implicated in catalysis and/or substrate binding (17, 31, 32). We previously investigated the importance of conserved ^163^DVD^165^ and ^221^FWGWRGEDDE^230^ motifs in the organization of the catalytic domain. Our data have highlighted the crucial role of Trp^224^ in substrate recognition and suggested a catalytic role for Asp^228^ (31). These findings were in accordance with the structural data from the recently solved crystal structure of the catalytic domain of *Drosophila melanogaster* dβ4GalT7 (33) and the human enzyme (34).

In the current study, we developed a structure-guided approach for the design of xyloside inhibitors of hβ4GalT7 that were tested on its galactosyltransferase activity *in vitro* and on GAG biosynthesis in cell assays. We explored the organization of the acceptor binding pocket, specifically probing the functional and structural contribution of a set of residues located in the vicinity of the catalytic center, and highlighted the crucial role of three tyrosine residues, *i.e.* Tyr^194^, Tyr^196^, and Tyr^199^, in the architecture of the acceptor substrate binding site and the creation of a hydrophobic environment. Based on these and previous findings, we synthesized compounds that incorporate critical structural elements both on the xylopyranoside and on the aglycone moieties to tightly bind the acceptor site of hβ4GalT7. This work revealed that the 4-deoxy-4-fluoro-Xyl linked to 4-methylumbelliferone (4-MU) strongly inhibited hβ4GalT7 activity *in vitro* and efficiently impaired GAG synthesis in a cell context. Such a compound will be a valuable tool for the exploration of GAG and PG synthesis and opens avenues toward the development of bioactive oligosaccharide structures for GAG biosynthesis regulation in a number of diseases implicating disorders of GAG synthesis.

## EXPERIMENTAL PROCEDURES

### 

#### 

##### Chemicals and Reagents

4-Methylumbelliferyl-β-d-xylopyranoside (4-MUX), UDP-α-d-Gal (UDP-Gal), and anti-goat IgG (whole molecule) peroxidase-conjugated antibody were provided from Sigma. Anti-Myc antibodies were from Invitrogen and anti-mouse IgG-peroxidase antibodies were purchased from Cell Signaling, whereas anti-decorin antibodies were from R&D Systems. Na_2_[^35^S]SO_4_ was from PerkinElmer Life Sciences. Cell culture medium was purchased from Invitrogen and restriction enzymes, T4 DNA ligase, and peptide *N*-glycosidase F from New England Biolabs. The eukaryotic expression vector pcDNA3.1(+) and competent One Shot® Top 10 *Escherichia coli* cells were provided by Invitrogen and the bacterial expression vector pET-41a(+) and *E. coli* BL21(DE3) cells were from Novagen-EMD Chemicals. The QuikChange site-directed mutagenesis kit was from Stratagene and the transfection agent ExGen 500 from Euromedex.

##### Chemical Synthesis

Naphthyl-4-deoxy-β-d-xylopyranoside (4H-Xyl-NP) (29) was obtained after protection of the 2,3 position of naphthyl-β-d-xylopyranoside by isopropylidene acetal followed by radical deoxygenation and deprotection. 4-Methylumbelliferyl-4-deoxy-β-d-xylopyranoside (4H-Xyl-MU) and 4-methylumbelliferyl-4-fluoro-β-d-xylopyranoside (4F-Xyl-MU) were synthesized from the reported starting material 4-methylumbelliferyl-2,3-di-*O*-benzoyl-β-d-xylopyranoside (35) by radical deoxygenation or stereocontrolled 4-fluorination followed by final deprotection (data not shown).

##### Molecular Modeling of the hβ4GalT7 Active Site in the Presence of 4-MUX and UDP-Gal

The crystal structure of hβ4GalT7 bound to UDP and to the manganese ion (PDB code 4IRQ) was used as template (34). The crystal structure of dβ4GalT7 (PDB code 4M4K), an inactive mutant (D211N) of dβ4GalT7 in complex with UDP-Gal, and xylobiose was superposed to the human enzyme structure, which was straightforward considering their strong sequence similarity (58% overall identity). Due to crystallization conditions, a Tris molecule is bound within the active site of the hβ4GalT7. When retrieved, it frees space within the cavity that can thus accommodate the Gal moiety. The coordinates of the Gal molecule from the dβ4GalT7 complex were merged to the UDP moiety of hβ4GalT7. This did not generate any steric clash within the active site. The resulting complex was then prepared using the Protein Preparation Wizard tool of the Schrödinger Suite (Schrödinger LLC, New York), with default settings (36). All water molecules were retrieved, except the one that coordinates the manganese ion. The hydrogen atoms were added to the protein and the ligand, ascribing a pH of 7.0. The histidine residues were treated as neutral. The selection of histidine enantiomers and the orientation of the asparagine and glutamine side chains were performed so as to maximize the hydrogen bond network. The partial atomic charges derived from the OPLS-2005 force field (37) were assigned to all ligand and protein atoms. Finally, an all-atom energy minimization with a 0.3 Å heavy-atom root mean square deviation criteria for termination was performed using the Impref module of Impact and OPLS-2005 (38). The 4-MUX ligand was prepared using the ligprep module (Schrödinger Release 2014–22014). The docking program Glide was used in Standard Precision mode, with OPLS-2005, to run rigid-receptor docking calculations (39, 40). The shape and physicochemical properties of the binding site were mapped onto a cubic grid with dimensions of 20 Å^3^ centered on the xylobiose. During the docking calculations, the parameters for van der Waals radii were scaled by 0.80 for receptor atoms with partial charges less than 0.15e. Ring conformational sampling was not allowed to maintain the ^4^C_1_ conformation of the Xyl ring, and no constraint was introduced. A maximum of 100 poses were retained and ranked according to the GlideScore scoring function. The best-docked pose of the 4-MUX ligand showed a root mean square deviation on the Xyl ring heavy atoms of 0.5 Å with the crystallographic xylobiose ligand, thus validating the docking protocol able to recover the position of this moiety.

##### Expression Vector Construction

The hβ4GalT7 sequence (GenBank® nucleotide sequence accession number NM_007255) was cloned by PCR amplification from a placenta cDNA library (Clontech), as previously described (41). For bacterial expression, a truncated form of hβ4GalT7 was expressed as a fusion protein with glutathione *S*-transferase (GST). The sequence lacking the codons of the first 60 N-terminal amino acids was amplified from the full-length cDNA and subcloned into NcoI and NotI sites of pET-41a(+) to produce plasmid pET-β4GalT7 (31). For the heterologous expression of hβ4GalT7 in mammalian cell lines, the full-length cDNA sequence was modified by PCR at the 5′ end to include a KpnI site and a Kozak consensus sequence, and at the 3′ end to include a sequence encoding a Myc tag and an XbaI site to be then subcloned into the KpnI-XbaI sites of the eukaryotic expression vector pcDNA3.1(+) to produce pcDNA-β4GalT7 as previously described (31). Mutations were constructed using the QuikChange site-directed mutagenesis kit, employing pcDNA-β4GalT7 or pET-β4GalT7 as template. Mutants were systematically checked by double strand sequencing. The human decorin cDNA sequence (GenBank accession number NM_001920.3) was cloned by PCR amplification from a placenta cDNA library (Clontech). For heterologous expression in eukaryotic cells, the full-length cDNA sequence was modified by PCR to include an AflII site, a Kozak consensus sequence at the 5′ end, a sequence encoding a His_5_ tag, and an XhoI site at the 3′ end. This sequence was subcloned into pcDNA3.1(+) to produce pcDNA-decorinHis as previously described (31).

##### Expression and Purification of the Soluble Form of hβ4GalT7

A single colony of *E. coli* BL21(DE3) cells transformed with the pET-β4GalT7 plasmid was cultured overnight at 37 °C in Luria broth (LB) medium containing 50 μg/ml of kanamycin. The overnight culture was transferred into fresh LB medium (1:100 dilution), supplemented with 50 μg/ml of kanamycin, and incubated at 37 °C until the *A*_600_ value reached 0.6–0.8. Expression of hβ4GalT7 was induced by addition of 1 mm isopropyl β-d-thiogalactopyranoside to the cell suspension, which was then incubated overnight at 20 °C under continuous shaking (200 rpm). The bacterial cells were then harvested by centrifugation at 7,000 × *g* for 10 min at 4 °C. The pellet was resuspended in Lysis buffer (50 mm sodium phosphate, 1 mm phenymethylsulfonyl fluoride, 1 mm EDTA, and 5% (v/v) glycerol, pH 7.4) supplemented with protease inhibitor mixture tablets (1 tablet/12 ml; Roche Diagnostics) and Benzonase® Nuclease (250 units/10 ml, Sigma). The suspended cells were then sonicated for 8 cycles of 30 s, at 30% power (Badelin Sonoplus GM70) with a 20-s interval on ice between each cycle. Soluble proteins were collected from the supernatant after centrifugation for 25 min at 12,000 × *g* and clarification by filtration (0.2 μm Supor® Membrane; PALL-Life Science). 10 ml of clarified extracts were applied onto a 1-ml glutathione-Sepharose High Performance column (GSTrap HP; GE Healthcare) connected to an AKTA prime plus instrument (GE Healthcare). Protein was eluted as 1-ml fractions using 50 mm Tris-HCl, pH 8.0, containing 10 mm reduced glutathione buffer. Protein purity of the eluted fractions was evaluated by 12% (w/v) SDS-PAGE analysis, followed by staining with Coomassie Brilliant Blue. Fractions containing the pure protein were used to determine the kinetic parameters of the enzyme. The same procedure was used for purification of the mutants. Protein concentration was measured using Quant-iT^TM^ assay kit and Qubit^TM^ spectrofluorimeter.

##### Determination of the in Vitro Kinetic Parameters of hβ4GalT7

The kinetic parameters *k*_cat_ and *K_m_* toward 4-MUX and UDP-Gal were determined as described (31). Briefly, 0.2 μg of purified wild-type or mutated GST-hβ4GalT7 were incubated for 30 min at 37 °C in a 100 mm sodium cacodylate buffer, pH 7.0, 10 mm MnCl_2_, with concentrations from 0 to 5 mm 4-MUX in the presence of fixed 1 mm UDP-Gal to determine the apparent *K_m_* toward 4-MUX, and with concentrations from 0 to 5 mm UDP-Gal in the presence of fixed 5 mm 4-MUX to determine the apparent *K_m_* toward UDP-Gal. The incubation mixture was then centrifuged at 10,000 × *g* for 10 min at 4 °C. The supernatant was analyzed by high performance liquid chromatography (HPLC) with a reverse phase C18 column (xBridge, 4.6 × 150 mm, 5 μm, Waters) using Waters equipment (Alliance Waters e2695) coupled to a UV detector (Shimadzu SPD-10A). Kinetic parameters were determined by nonlinear least squares regression analysis of the data fitted to the Michaelis-Menten rate equation using the curve-fitter program of Sigmaplot 9.0 (Erkraft, Germany).

##### In Vitro Competition Assays of hβ4GalT7 Activity by C4-modified Xylosides

The *in vitro* inhibition assays of the wild-type GST-hβ4GalT7 were carried out using 0.2 μg of purified protein incubated for 30 min at 37 °C in a 100 mm sodium cacodylate buffer, pH 7.0, 10 mm MnCl_2_, with 0.5 mm 4-MUX and 1 mm UDP-Gal, in the presence of concentrations from 0 to 5 mm 4H-Xyl-NP, 4H-Xyl-MU, or 4F-Xyl-MU. Quantification of the reaction product was carried out by HPLC, as described above. The enzyme activities were reported as a function of the logarithmic values of inhibitor concentration. IC_50_ values were determined by fitting the experimental dose-response curves using the curve-fitter program of Sigmaplot 9.0 (Erkraft, Germany). *K_i_* values were calculated from IC_50_ values according to the Cheng-Prusoff's equation (42, 43).

##### In Cellulo Analysis of GAG Chains Biosynthesis by Na_2_[^35^SO_4_] Incorporation

GAG chains biosynthesis using 4-MUX as primer substrate was determined with CHOpgsB-618 cells (American Type Culture Collection). Cells were cultured in Dulbecco's modified Eagle's medium/F-12 (DMEM/F-12) (1:1), supplemented with 10% fetal bovine serum (Dutscher), penicillin (100 units/ml)/streptomycin (100 mg/ml), and 1 mm glutamine, then transfected with the wild-type or mutant pcDNA-β4GalT7-Myc plasmid or with the empty pcDNA3.1 vector at 70% cell confluence. Transfected cells were then incubated in low sulfate medium (Fisher) supplemented with 10 μCi/ml of Na_2_[^35^SO_4_] in the presence of 0.5 or 10 μm 4-MUX for 16 h. For GAG chain isolation 1 ml of culture medium was applied to a G-50 column (GE Healthcare) to separate radiolabeled GAG chains from the non-incorporated Na_2_[^35^SO_4_] and radiolabeling was quantified by scintillation counting. In parallel, the hβ4GalT7 expression level was checked by Western blotting using a primary anti-Myc (1/5,000) and a secondary anti-mouse antibody (1/10,000). To test the inhibitory potency of C4-modified xylosides, the molecules were added at 0 to 100 μm concentration together with 4-MUX (5 μm) for 16 h prior to isolation and quantification of radiolabeled GAGs. To test the cytotoxicity of xyloside inhibitors in CHOpgsB-618 cells expressing the wild-type hβ4GalT7, cells were seeded at 150,000 cells/well in 12-well plates, and incubated for 48 h at 37 °C in the presence of 0 to 400 μm inhibitor, or 4-MUX as a control. The ratio of viable cells upon the total number of cells was determined using the cell counter TC20 (Bio-Rad) in the presence of a vital marker (trypan blue).

##### In Cellulo Analysis of Decorin Core Protein Glycosylation

CHOpgs-B618 cells stably transfected with pcDNA-decorinHis encoding the human decorin core protein (31, 44) were transiently transfected with pcDNA3.1 or with recombinant vector encoding either the wild-type or mutated hβ4GalT7-Myc as described above. 48 Hours following transfection, the cell medium was collected, concentrated by centrifugation at 4 °C for 15 min at 3000 × *g*, using the Amicon Ultracell 30 MWCO concentrating system (Merck Millipore, Germany), and submitted to SDS-PAGE (25 μg of protein/well). The glycosylation level of the decorin core protein was monitored by immunoblot using a 1/5,000 dilution of primary polyclonal anti-human decorin antibody (VWR) and a 1/10,000 dilution of secondary anti-goat antibody coupled to horseradish peroxidase (Sigma), then quantified using ImageJ software. Briefly, the level of decorin glycosylation was expressed as relative band intensity by normalizing the band intensity value for the glycosylated form upon the total intensity value for the bands corresponding to the glycosylated and non-glycosylated forms of decorin core protein. The expression level of the decorin core protein in pcDNA3.1-transfected cells was used as the negative control and served as a loading control. On the other hand, the level of glycosylated decorin in cells expressing wild-type hβ4GalT7 was used as positive control for decorin glycosylation.

## RESULTS

### 

#### 

##### Molecular Modeling of the hβ4GalT7 Acceptor Binding Site

**FIGURE 1.**
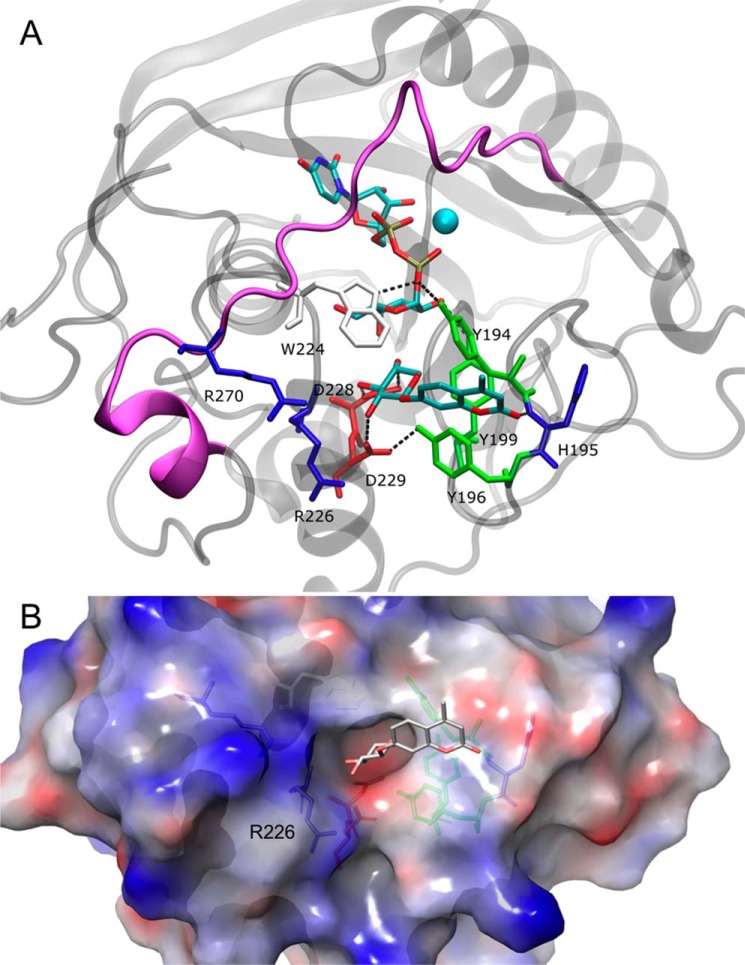
**Molecular modeling of the hβ4GalT7 structure.** *A,* view of the active site of hβ4GalT7 in complex with donor (*UDP-Gal*) and acceptor (*4-MUX*) substrates. The protein α-carbon trace is represented as *gray ribbons*; the *purple ribbon* corresponds to the protein backbone that is not seen in the open conformation. *B,* surface representation of the acceptor binding site, in the same orientation as in *A*. The electrostatic potential is mapped onto the protein surface and is colored from *red* (negative) to *blue* (positive).

In the present study, we aimed to identify amino acids important for structural organization of the hβ4GalT7 acceptor substrate binding site. We took advantage of the recent crystal structure of hβ4GalT7 in complex with UDP (34) to build a molecular model of this enzyme in complex with both the sugar donor UDP-Gal and the acceptor 4-MUX (Fig. 1*A*). The modeled structure is in a closed conformation, considered to be the catalytically competent form, and the hydrogen bond network around the UDP moiety is fully conserved. We first examined the position of a series of tyrosine residues, *i.e.* Tyr^194^, Tyr^196^, and Tyr^199^, that were suggested to be involved in binding of the xylobiose in the dβ4GalT7 structure (33). Our computational analysis indicates that Tyr^194^ stabilizes both the donor and acceptor substrates location by establishing a hydrogen bond with a β-phosphate oxygen of UDP-Gal and a π-stacking interaction with the 4-methylumbelliferyl moiety, respectively (Fig. 1*A*). Residue Tyr^196^ is not hydrogen bonded to the substrates but to the side chain of residue Asp^229^, allowing its second carboxylic oxygen to be suitably oriented to form a hydrogen bond with the O2 atom of Xyl. The spatial orientation of Tyr^199^ inside the substrate binding pocket allows the formation of a hydrogen bond between its side chain hydroxyl and the O2 atom of the Gal moiety of UDP-Gal. Altogether, residues Tyr^194^, Tyr^196^, and Tyr^199^ form a strongly hydrophobic cluster that is required for correct binding of the substrates.

Analysis of the His^195^ position, a conserved amino acid located between the two active site Tyr^194^ and Tyr^196^ residues, shows no hydrogen bond involving its side chain. However, the backbone nitrogen atom of this residue is hydrogen bonded with the CO group of 4-MUX (Fig. 1*A*). As illustrated in Fig. 1*B*, Arg^226^ is located on the surface of the acceptor binding site contributing to an amphipathic entry door with the aromatic residues. In our model, there is no hydrogen bond involving the side chain of Arg^226^. Instead, its backbone nitrogen atom is hydrogen bonded with the O3 atom of the Xyl moiety of 4-MUX (Fig. 1*A*).

The structural impact of Arg^270^ on enzyme activity, in the context of EDS was also addressed. The model structure of hβ4GalT7 reveals that Arg^270^ belongs to the flexible loop (261–284) that moves upon donor substrate binding, thus creating the acceptor substrate binding site (Fig. 1*A*). This conformational change leads to the closed and catalytically competent conformation of the active site. However, the crystal structure of the human enzyme (34), as well as our own model in complex with both the donor and acceptor substrates do not highlight specific interactions established by this residue, although its close location to the surface of the active site has to be underlined (Fig. 1*B*).

##### Kinetic Properties of the Human Recombinant hβ4GalT7 Mutants Expressed in E. coli

To assess the functional importance of the residues of the acceptor binding site highlighted by our model, we carried out point mutagenesis and analyzed the consequences of conservative and non-conservative mutations on the kinetic parameters of hβ4GalT7 expressed and purified from recombinant *E. coli* cells. The wild-type enzyme and engineered mutants were produced as truncated fusion proteins lacking the 60 N-terminal amino acids (including the transmembrane domain and part of the stem region) linked to GST and purified by affinity chromatography (data not shown). This led to 1.0 to 2.5 mg of pure protein per liter of culture for wild-type and mutant hβ4GalT7. Kinetic assays were performed using 4-MUX as acceptor substrate, which allowed quantification of the transfer reaction product by UV detection coupled to HPLC. The *k*_cat_ and *K_m_* values of the wild-type enzyme toward UDP-Gal and 4-MUX shown in Table 1 were in agreement with previous work (17, 31).

**TABLE 1:** **Kinetic parameters of wild-type and mutant GST-β4GalT7** Kinetic parameters towards donor (UDP-Gal) and acceptor (4-MUX) substrates were determined as described under “Experimental Procedures.” The results are the mean values of three independent determinations ± S.D. on assays performed in duplicate.

Enzyme	*k*_cat_	UDP-Gal		4-MUX	
		*K_m_*	*k*_cat_/*K_m_*	*K_m_*	*k*_cat_/*K_m_*
	*min*^−*1*^	*mm*	*min*^−*1*^·*mm*^−*1*^	*mm*	*min*^−*1*^·*mm*^−*1*^
GST-β4GalT7	90.5 ± 2.3	0.22 ± 0.02	425	0.35 ± 0.02	250
Y194A	ND*^a^*	ND		ND	
Y194F	ND	ND		ND	
H195A	115.9 ± 9.7*^b^*	0.40 ± 0.02*^b^*	291	0.64 ± 0.02*^b^*	180
H195Q	97.4 ± 2.4*^b^*	0.33 ± 0.02*^b^*	295	0.55 ± 0.02*^b^*	177
H195R	89.6 ± 1.3	0.32 ± 0.02*^b^*	295	0.35 ± 0.03	242
Y196A	ND	ND		ND	
Y196F	30 ± 1.1*^b^*	0.34 ± 0.06*^b^*	88	1.06 ± 0.06*^b^*	28
Y199A	ND	ND		ND	
Y199F	72.3 ± 5.8*^b^*	0.32 ± 0.02*^b^*	243	0.59 ± 0.06*^b^*	113
R226A	53.6 ± 2.0*^b^*	0.34 ± 0.03*^b^*	171	0.44 ± 0.05*^b^*	112
R226K	81.1 ± 2.9*^b^*	0.29 ± 0.02*^b^*	282	0.46 ± 0.01*^b^*	175
R270A	46.4 ± 0.2*^b^*	0.27 ± 0.01*^b^*	184	0.60 ± 0.02*^b^*	72
R270K	48.7 ± 1.5*^b^*	0.37 ± 0.02*^b^*	139	0.54 ± 0.07*^b^*	85

*^a^* ND indicates that no kinetic constant could be determined using excess acceptor or donor substrate.

*^b^* The results were analyzed with Student's *t* test and considered as significant when *p* < 0.05.

Substitution of Tyr^194^ by alanine led to an inactive enzyme, and its conservative substitution by phenylalanine did not restore the galactosyltransferase activity of hβ4GalT7 (Table 1), indicating a critical role of this residue and, importantly, of the hydroxyl group of the tyrosine side chain. The mutation of Tyr^196^ to alanine totally abolished enzyme activity, whereas replacement of this residue by phenylalanine led to a slightly active enzyme. The Y196F mutation did not impair enzyme affinity toward the donor substrate to a major extent but this mutant presented a lower affinity toward 4-MUX with a *K_m_* value about 3-fold that of the wild-type enzyme (Table 1). As observed in the case of Tyr^194^ and Tyr^196^, the non-conservative mutation Y199A led to a total loss of enzyme activity. However, similarly with what was observed for Tyr^196^, substitution of Tyr^199^ by phenylalanine led to an active hβ4GalT7 enzyme with *K_m_* values toward UDP-Gal and 4-MUX and a *k*_cat_ value only weakly affected compared with the wild-type enzyme. These data suggest that the aromatic ring of phenylalanine at position 199 is sufficient to support xyloside binding and activity.

Substitution of His^195^ by alanine, glutamine, or arginine was carried out. The *K_m_* values of all three mutants toward UDP-Gal and 4-MUX were mostly comparable with those of the wild-type enzyme, indicating that these mutations had no major effect upon hβ4GalT7 affinity toward its substrates. Moreover, the substitutions at position 195 did not affect the rate of reaction transfer, as indicated by the *k*_cat_ values that were essentially unchanged (Table 1). Altogether, these results indicate that the side chain of His^195^ does not play a critical role in xyloside binding and hβ4GalT7 catalytic activity. Substitution of Arg^226^ by alanine or lysine did not impair the affinity toward the substrates with *K_m_* values for UDP-Gal and 4-MUX, which were in the same range to that of the wild-type enzyme, and produced a moderate decrease (about 2-fold) of the catalytic constant value (Table 1).

The Arg^270^ residue is mutated to cysteine in the progeroid form of the EDS syndrome, and we previously showed that this mutation led to a significant decrease in hβ4GalT7 activity, mainly due to a reduced affinity toward 4-MUX (about 10-fold, see Ref. 17). To ascertain the contribution of this residue in hβ4GalT7 activity and xyloside binding, we performed kinetic assays following the conservative R270K and non-conservative R270A mutations. The *k*_cat_ values for both mutants were about two times lower than that found for the wild-type enzyme and the *K_m_* value toward UDP-Gal was almost unaffected (Table 1).

##### Effect of Tyr^194^, Tyr^196^, Tyr^199^, His^195^, Arg^226^, and Arg^270^ Mutations on the Galactosyltransferase Activity of hβ4GalT7 Toward 4-MUX in Cellulo

**FIGURE 2.**
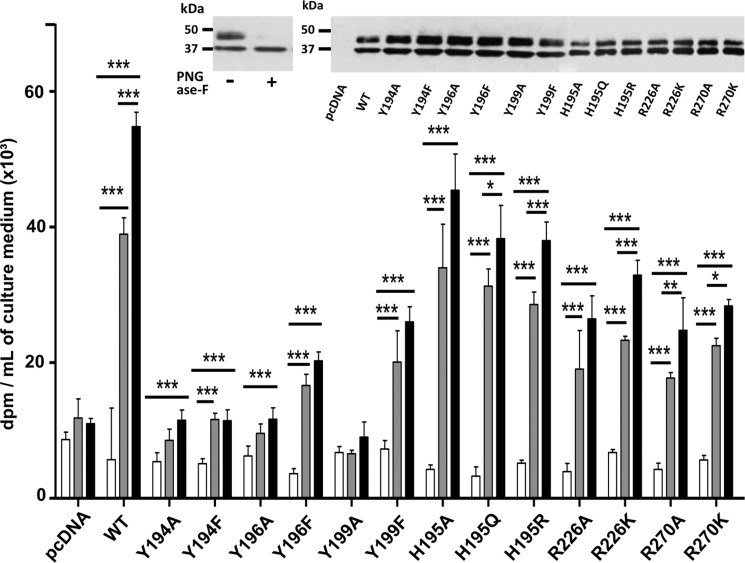
**Effect of wild-type and mutated hβ4GalT7 expression on GAG chains primed from 4-MUX in CHOpgsB-618 cells.** Cells were transiently transfected with wild-type (WT) or mutated hβ4GalT7 cDNA or with empty vector (*pcDNA*), and GAG chains synthesis was quantified by scintillation counting following Na_2_[^35^SO_4_^2−^] incorporation, using 0 (*white bars*), 5 (*gray bars*), and 10 μm (*black bars*) 4-MUX. Immunoblot analyses of the protein expression level in CHOpgsB-618 cells transfected with the vector coding for the wild-type or mutated hβ4GalT7 are shown as the *inset*. The enzyme was identified at the band of ∼35 kDa, whereas the upper band corresponding to ∼39 kDa band could be attributed to the *N*-glycosylated enzyme as demonstrated by its disappearance upon addition of peptide *N*-glycosidase F (*PNGase F*) (*left panel*). Both bands intensities were used to quantify the total protein expression level (ImageJ software). The immunoblot analysis indicates that the mutated enzymes were all expressed at a comparable level to that of the wild-type hβ4GalT7 (*right panel*). Data are mean ± S.E. of three independent experiments performed in triplicate. Statistical analysis was carried out by the Student's *t* test with *, *p* < 0.05; **, *p* < 0.01; and ***, *p* < 0.001 *versus* GAG synthesis in the absence of 4-MUX.

To check the importance of the selected residues on the hβ4GalT7 function in a cellular context, we designed an experimental procedure involving CHOpgsB-618 cells transfected with hβ4GalT7 cDNA encoding either the wild-type or mutant enzymes fused to a Myc tag sequence at their C-terminal end, as described under “Experimental Procedures.” CHOpgsB-618 cells expressing the recombinant enzymes were tested for *in cellulo* galactosyltransferase activity in the absence or presence of 4-MUX as the exogenous acceptor substrate. The expression level of enzymes was checked by immunoblot analysis using ImageJ software. As shown in Fig. 2, an additional upper band at ∼39 kDa was observed (Fig. 2, *inset*). This ∼39-kDa band can be attributed to the *N*-glycosylated form of the enzyme as it disappears upon peptide *N*-glycosidase F digestion (Fig. 2, *inset*, *left panel*). Both bands were taken into account to quantify the total enzyme expression level. The results indicate that all mutants considered in these experiments were expressed at a similar level to that of the wild-type protein (Fig. 2, *inset*, *right panel*). As shown in Fig. 2, the GAG synthesis level in cells expressing wild-type hβ4GalT7 was about 7- and 9.5-fold higher in the presence of 5 and 10 μm 4-MUX, respectively, than in the absence of acceptor substrate, indicating that CHOpgsB-618 cells expressing hβ4GalT7 are able to prime efficiently GAG chains synthesis from 4-MUX, in agreement with previous studies (17, 31).

As expected, cells expressing either Y194A or Y194F mutant in the presence of 4-MUX showed GAG synthesis levels comparable with those obtained with cells transfected with empty vector (Fig. 2). These *in cellulo* assays confirm that any mutation affecting the Tyr^194^ position leads to a total loss of hβ4GalT7 activity. Substitution of Tyr^196^ to alanine dramatically reduced the GAG synthesis rate whose level in the presence of 4-MUX was comparable with that obtained with cells transfected with empty vector. This is consistent with the loss of enzymatic activity observed in the *in vitro* assays (Table 1). By contrast, the conservative mutation Y196F allowed GAG chain priming from 4-MUX. However, the GAG expression level reached with this mutant was about 2–3-fold lower than that of the wild-type enzyme, at 4-MUX at 5 and 10 μm concentrations, respectively (Fig. 2). This result is consistent with the drastic decrease of the *k*_cat_/*K_m_* value toward 4-MUX found for the purified Y196F mutant. Comparable results were obtained with cells expressing hβ4GalT7 whose sequence is mutated on the Tyr^199^ position. Indeed, cells expressing the Y199A mutant were unable to synthesize GAG chains from 4-MUX, whereas cells expressing Y199F showed GAG chain synthesis at a level about half of that observed with cells expressing wild-type enzyme (Fig. 2). These results are in line with the reduced efficiency exhibited *in vitro* by the enzyme substituted on the Tyr^199^ position (Table 1). Altogether, these results demonstrate that both conservative and non-conservative mutations affecting Tyr^194^, Tyr^196^, or Tyr^199^ significantly impaired GAG chains biosynthesis in a cellular context, in line with *in vitro* data (Table 1).

In the presence of 4-MUX, the GAG synthesis rate in cells expressing H195A, H195Q, and H195R mutants was moderately reduced, *i.e.* 10 to 15% lower than that of cells expressing the wild-type enzyme (Fig. 2). These results indicate that the side chain of this residue does not influence galactosyltransferase activity of hβ4GalT7 in the context of 4-MUX-primed GAG chains in eukaryotic cells, corroborating the findings that none of the mutations of His^195^ significantly affect *in vitro* activity (Table 1). The level of [^35^SO_4_^2−^] incorporation in the presence of 4-MUX in cells expressing R226A was about 2 times lower than that of the wild-type enzyme (Fig. 2). In addition, replacement of Arg^226^ by lysine slightly increased the GAG expression level compared with alanine substitution, reaching about 60% that obtained with the wild-type, at 5 and 10 μm 4-MUX. Corroborating *in vitro* kinetic parameters, these cellular assays indicate that modification of the side chain of Arg^226^ produces minor effects on galactosyltransferase activity.

We finally examined the impact of mutations of the Arg^270^ residue upon GAG synthesis in eukaryotic cells. We observed that the GAG synthesis rate in cells expressing the R270A mutant was about 55% lower than that of the wild-type enzyme, at 5 and 10 μm 4-MUX concentration (Fig. 2). The GAG synthesis level of the conservative mutant R270K was also about 2-fold reduced compared with the wild-type (Fig. 2). These results confirm that mutations of Arg^270^ significantly affect the capacity of hβ4GalT7 to synthesize GAG chains from 4-MUX in a cellular context.

##### Effect of Tyr^194^, Tyr^196^, Tyr^199^, His^195^, Arg^226^, and Arg^270^ Mutations on the Ability of hβ4GalT7 to Initiate the Glycosylation of the Decorin PG in Cellulo

**FIGURE 3.**
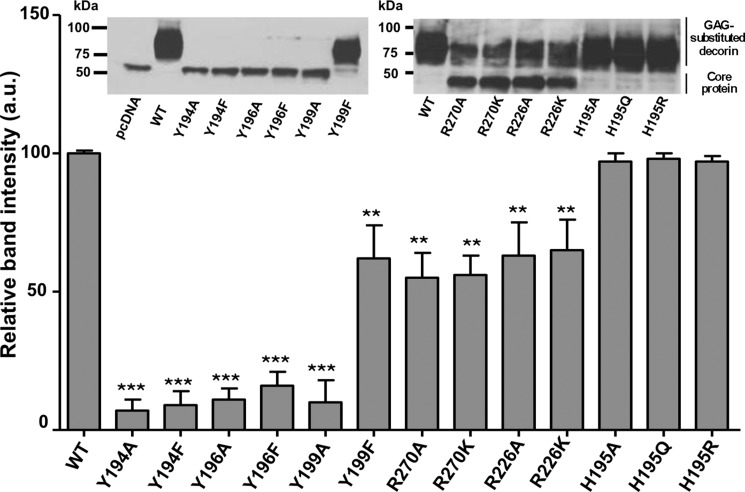
**Effect of wild-type and mutated hβ4GalT7 expression on decorin core protein glycosylation in CHOpgsB-618 cells.** Cells stably expressing the human recombinant decorin core protein were transfected with the recombinant vector encoding either the wild-type (WT) or mutated hβ4GalT7. The decorin glycosylation level was monitored by immunoblot then quantified using ImageJ software, as described under “Experimental Procedures.” Immunoblot analyses of the decorin core protein glycanation level are shown as inserts for CHOpgsB-618 cells transfected with empty (*pcDNA*) or with the recombinant vector coding for WT, Y194A, Y194F, Y196A, Y196F, Y199A, or Y199F hβ4GalT7 (*left panel*), and for WT, R270A, R270K, R226A, R226K, H195A, H195Q, or H195R hβ4GalT7 (*right panel*). The band observed at a molecular mass of ∼35 kDa can be attributed to the decorin core protein, whereas the wide upper band corresponding to a molecular mass ≥75 kDa corresponds to the glycosylated decorin. Data are mean ± S.E. from three independent experiments performed in triplicate. Statistical analysis was carried out by the Student's *t* test with **, *p* < 0.01 and ***, *p* < 0.001 *versus* decorin glycosylation in cells expressing the wild-type hβ4GalT7.

We next determined whether the mutations would affect GAG chain formation on the core protein of decorin, used as a model PG (31). To this aim, CHOpgsB-618 cells were engineered to stably express the recombinant human decorin, and were transiently transfected with a pcDNA3.1 vector encoding the wild-type or mutant forms of hβ4GalT7. This allowed monitoring of the GAG substitution of the secreted PG by Western blot analysis (Fig. 3, *inset*, *left* and *right panels*). The rates of decorin PG glycosylation in cells expressing wild-type or mutant hβ4GalT7 were determined as described under “Experimental Procedures,” then reported onto the histogram (Fig. 3). Results showed that non-conservative mutations of the tyrosine residues at positions 194, 196, and 199 as well as mutations of Tyr^194^ and Tyr^196^ to phenylalanine fully abolished the glycosylation of decorin (Fig. 3). These data were consistent with the drop of GAG chains primed from 4-MUX in cells expressing hβ4GalT7 mutated at these positions (Fig. 2). However, the substitution of Tyr^196^ to phenylalanine induced a more dramatic effect on the glycosylation of decorin than on the *in vitro* or *in cellulo* activity toward 4-MUX. Furthermore, results shown in Fig. 3 indicate that conservative mutation Y199F allowed recovery up to 70% of the decorin glycosylation level compared with cells expressing the wild-type hβ4GalT7.

We also assessed the role of the His^195^ residue in the glycosylation process of decorin. The results shown in Fig. 3 indicate that none of the mutations, H195A, H195Q, or H195R, significantly affected the level of decorin glycosylation. These data are consistent with the results obtained on the *in vitro* and *in cellulo* activity of the enzyme toward 4-MUX. Together, mutagenesis experiments indicate that modification of the amino acid side chain at position 195 did not greatly affect xyloside binding and galactosyltransferase activity of hβ4GalT7. Investigation of the effect of the Arg^226^ mutation upon the ability of CHOpgsB-618 cells to glycosylate decorin showed that the glycosylation level reached with cells expressing R226A or R226K was about 65% of that obtained with cells expressing the wild-type enzyme. These results were consistent with the *in vitro* and *in cellulo* GAG chain synthesis assays (Fig. 3).

Because we aimed to better understand the molecular basis of the EDS syndrome, it was important to further investigate the impact of mutations affecting the Arg^270^ position upon the decorin glycosylation. The decorin glycosylation level reached with cells expressing either R270A or R270K mutant was about half of that obtained with cells expressing the wild-type hβ4GalT7, consistent with *in vitro* and *in cellulo* galactosyltransferase assays (Fig. 3).

##### Xyloside Inhibitors Design and in Vitro and in Cellulo Competition Assays

**FIGURE 4.**
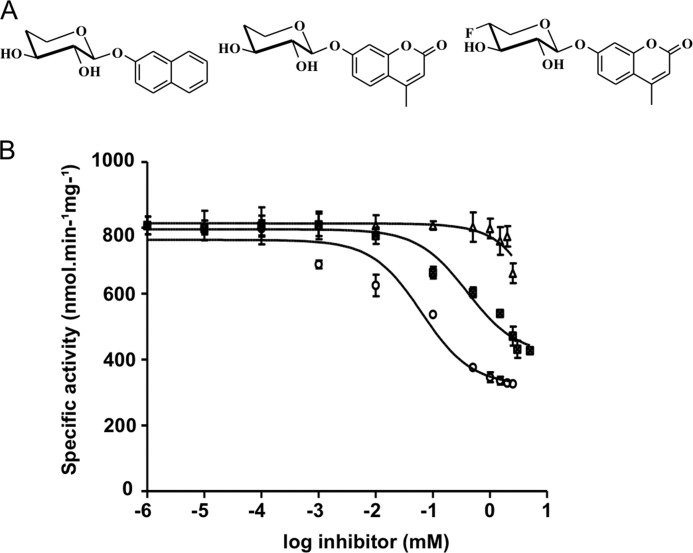
**Inhibitory effect of C4-modified xylosides on hβ4GalT7 activity.** *A*, chemical structures of the xyloside analogs synthesized and tested as inhibitors. From left to right: 4H-Xyl-NP, 4H-Xyl-MU, and 4F-Xyl-MU. *B,* inhibition assays using purified recombinant wild-type hβ4GalT7 in the presence of fixed 4-MUX (0.5 mm) and UDP-Gal (1 mm). Activities are presented as function of the logarithm of increasing inhibitor concentrations (0–5 mm); 4H-Xyl-NP (▴), 4H-Xyl-MU (■), and 4F-Xyl-MU (○). Results are the mean ± S.E. of three independent determinations on assays performed in duplicate.

We next took advantage of the knowledge gained from our investigation of the organization of the acceptor substrate binding site to synthesize and test xyloside analogs as potential inhibitors of hβ4GalT7. To this end, *in vitro* competition assays were performed as described under “Experimental Procedures.” The specific activity as a function of the logarithm values of the inhibitor concentrations are reported in Fig. 4*B* and data fitted to the logistic equation provided IC_50_ values as reported in Table 2.

**TABLE 2:** **Kinetic inhibition parameters of hβ4GalT7 with C4-modified xylosides** IC_50_ and *K_i_* values are the values of three independent experiments mean ± S.D. on assays performed in duplicate.

Xylosides	IC_50_	*K_i_*
	*mm*	
4H-Xyl-NP	ND*^a^*	ND
4H-Xyl-MU	1.28 ± 0.22	0.53 ± 0.10
4F-Xyl-MU	0.06 ± 0.02	0.03 ± 0.01

*^a^* ND, not determined.

Because the C4-position is critical for both binding and transfer of the Gal residue from UDP-Gal onto the xyloside acceptor, we first synthesized a 4-deoxy derivative of 4-MUX (4H-Xyl-MU, Fig. 4*A*) and tested this compound as inhibitor of hβ4GalT7 *in vitro*. 4H-Xyl-MU was able to inhibit up to 50% of the initial activity at a 2 mm concentration (Fig. 4*B*), with an IC_50_ value of about 1 mm and a *K_i_* value of about 0.5 mm (Table 2). To test whether hydrogen bond formation between 4-MUX and the protein via His^195^ is important for the inhibitory potency, we synthesized 4H-Xyl-NP, which the aglycone structure is unable to establish such an interaction, and compared its inhibitory effect to 4H-Xyl-MU. This compound produced a decrease of hβ4GalT7 activity toward 4-MUX less than 25% at the highest concentration (Fig. 4*B*) that did not allow determining IC_50_ and *K_i_* values. These results clearly indicated that 4H-Xyl-NP is a weak inhibitor of hβ4GalT7. We next substituted the equatorial hydrogen of the C4 atom of the Xyl moiety by a fluorine atom, closer to oxygen in terms of electronegativity and predicted to fit the active site in terms of steric hindrance. 4F-Xyl-MU led up to 60% inhibition of the hβ4GalT7 activity at the highest concentration (Fig. 4*B*), with an IC_50_ of 0.06 mm and a *K_i_* of 0.03 mm. The inhibition constant for this compound is more than 10 times lower than that reached for the deoxy analog (Table 2).

**FIGURE 5.**
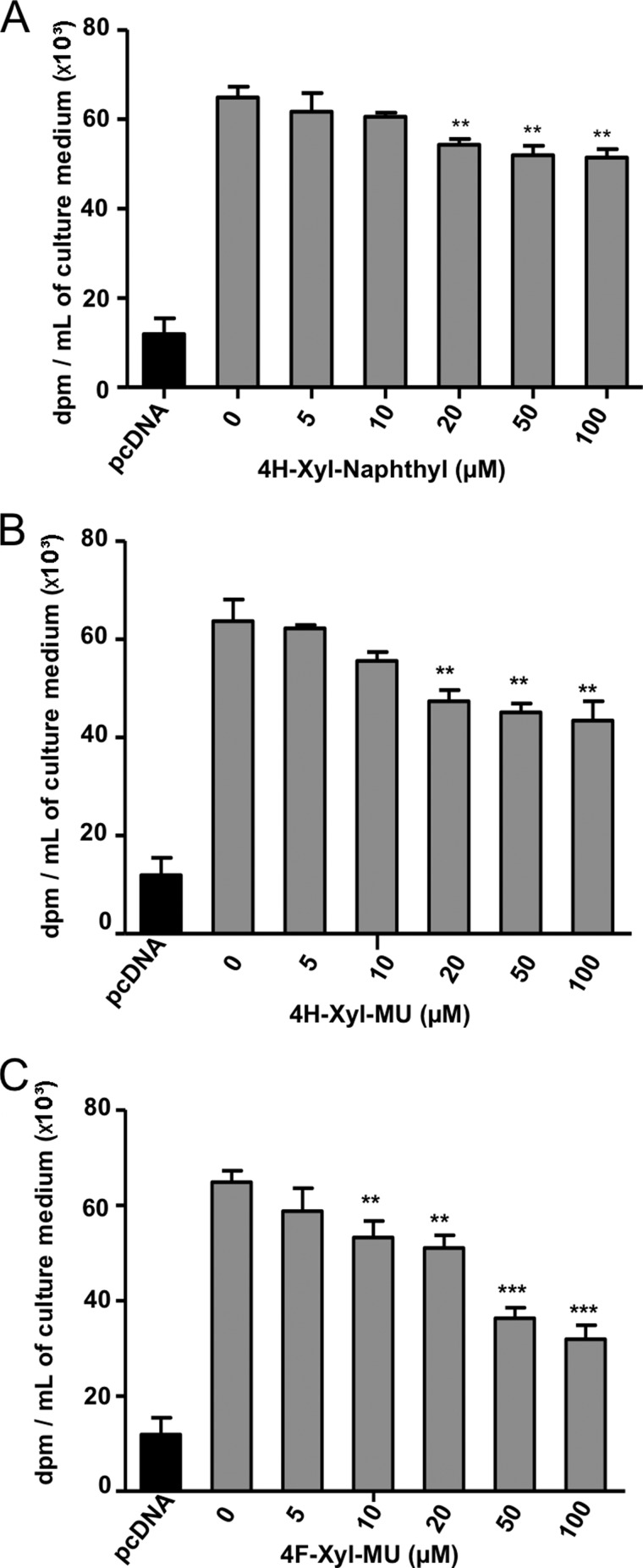
**Inhibitory effect of C4-modified xylosides upon 4-MUX-primed GAG chains synthesis in CHOpgsB-618 cells expressing the recombinant wild-type hβ4GalT7.** CHOpgsB-618 cells transiently transfected with wild-type hβ4GalT7 cDNA were incubated with 5 μm 4-MUX and Na_2_[^35^SO_4_^2−^], in the presence of 4H-Xyl-NP (*panel A*), 4H-Xyl-MU (*panel B*), and 4F-Xyl-MU (*panel C*). The GAG expression level in cells transfected by the empty pcDNA vector was taken as negative control. Results are the mean ± S.E. of three independent experiments performed in triplicate. Statistical analysis was carried out using the Student's *t* test with **, *p* < 0.01 and ***, *p* < 0.001 v*ersus* GAG synthesis rate in the absence of inhibitor.

To complement the *in vitro* assay, we assessed the ability of the synthesized xyloside derivatives to inhibit GAG chain biosynthesis *in cellulo*. Addition of 4H-Xyl-NP produced a moderate but significant 20% decrease of GAG chain synthesis in CHOpgsB-618 cells, when used at 50 and 100 μm (Fig. 5*A*). This correlated with the weak *in vitro* inhibition level obtained with this compound. The compound 4H-Xyl-MU allowed a larger inhibition of GAG chain synthesis, with up to 30% reduction of the GAG synthesis rate at similar concentrations (Fig. 5*B*). The best inhibitory effect was observed when performed in the presence of 4F-Xyl-MU leading to up to 50% inhibition of the initial GAG chain synthesis rate at 100 μm concentration (Fig. 5*C*). Interestingly, preliminary results indicated that 400 μm 4F-Xyl-MU inhibited the initial decorin glycosylation rate by about 50%, without affecting the viability of CHOpgsB-618 cells (data not shown). Altogether, the latter data confirmed that this fluorinated compound should be considered as a promising non-cytotoxic xyloside-based inhibitor of hβ4GalT7.

## DISCUSSION

β4GalT7 is a unique enzyme in the GAG biosynthetic pathway with regard to its capacity to use exogenous xyloside molecules as substrates and/or inhibitors that can efficiently modulate GAG synthesis *in vitro* and *in vivo* (19, 20, 45). This glycosyltransferase is also central in the GAG synthesis process because the formation of the tetrasaccharide linker is a prerequisite to the polymerization of both heparan sulfate and chondroitin/dermatan sulfate chains. The human enzyme thus represents a prime target for the design of effectors of GAG synthesis as drugs to correct GAG disorders associated with numerous malignant conditions such as genetic diseases and cancer. To meet this challenge, we pioneered structure-function studies of the recombinant hβ4GalT7. We previously carried out structural, thermodynamic, and phylogenetic investigations that identified key amino acid residues mainly implicated in the recognition and binding of the donor substrate (31, 46). We also provided insight into the molecular basis of the GAG defects characterizing rare forms of EDS syndrome (17, 47). In the present work, to develop xyloside compounds that will specifically target the hβ4GalT7 activity for a therapeutic purpose, we explored the architecture of the acceptor substrate binding site. To this aim, we combined functional investigations including site-directed mutagenesis, kinetic analyses, *in vitro* and *in cellulo* evaluation of galactosyltransferase activity and GAG synthesis, and a computational approach. This allowed mapping the acceptor binding site and to design and synthesize a potent xyloside-based inhibitor of GAG synthesis.

We first targeted a set of three tyrosine residues, Tyr^194^, Tyr^196^, and Tyr^199^, as well as His^195^ belonging to the same conserved motif, that occupy a strategic position surrounding the xyloside acceptor substrate (Ref. 34 and the present data). Our mutational analysis led to a remarkable observation because alanine substitution of each of these tyrosine completely abolished hβ4GalT7 activity. The tyrosine-alanine mutants (i) were devoid of galactosyltransferase activity *in vitro*, (ii) were unable to prime GAG synthesis from 4-MUX *in cellulo*, and (iii) did not promote decorin glycosylation, thus supporting a prominent function of this set of aromatic residues. Mutating Tyr^194^, Tyr^196^, and Tyr^199^ with phenylalanine revealed that the substitution differently affected hβ4GalT7 activity depending on the position. Noteworthy, the presence of the hydroxyl group of Tyr^194^ was indispensible because the conservative Y194F mutant completely lacked *in vitro* or *in cellulo* galactosyltransferase activity toward 4-MUX and was unable to sustain glycosylation of decorin. This observation is likely to be explained by a functionally important interaction between the hydroxyl group of this tyrosine and the β-phosphoryl group of UDP-Gal that was observed in all structures and models of β4GalT7 (Ref. 34 and this report, see Fig. 1). Most importantly, our computational model and experimental data suggested that the critical role of Tyr^194^ also arises from a stacking interaction between the aromatic ring of this residue and the 4-methylumbelliferyl moiety of 4-MUX. Altogether, these data indicate that Tyr^194^ occupies a strategic location in the catalytic center and interacts with both the donor nucleotide and the aglycone group of 4-MUX. In the case of Tyr^196^, the presence of the hydroxyl group of the tyrosine residue was also a major structural element because its replacement by phenylalanine only slightly restored the activity toward 4-MUX *in vitro* and *in cellulo*. The Y196F mutant did not sustain decorin glycosylation, also supporting an important role of this residue in the glycosylation of endogenous proteoglycans. Our model provides a molecular explanation to these results, because it shows that Tyr^196^ is not directly involved in the binding of the acceptor substrate but rather forms a hydrogen bond between its hydroxyl group and Asp^229^, this latter residue establishing an important interaction with O2 of the Xyl moiety. This supports the idea that interactions with the hydroxyl in the C2 position control a strict geometry via both Asp^229^ and Tyr^196^, in agreement with the physiologically important regulatory role of Xyl-phosphate substitution in position 2 on GAG synthesis (41, 48). Furthermore, our model suggests that this residue is part of the cavity floor in agreement with structural data indicating that the acceptor substrate xylobiose is located in a hydrophobic binding pocket formed by Tyr^177^, Tyr^179^, and Trp^207^ in the *Drosophila* structure, and by Tyr^194^, Tyr^196^, and Trp^224^ in hβ4GalT7. The present data complements our previous findings demonstrating that Trp^224^ is a crucial active site residue (31). Differently to the preceding studied tyrosines, hβ4GalT7 tolerated well the substitution of tyrosine to phenylalanine at position 199 leading to a mutant that was active toward 4-MUX *in vitro* and was able to prime GAG synthesis from 4-MUX and onto the decorin core protein. Consistently, Tyr^199^ is substituted by a phenylalanine in the *Drosophila* enzyme, suggesting that the presence of a hydrophobic aromatic ring is sufficient at this position. Further analysis of our molecular model predicts hydrogen bond formation between the tyrosine hydroxyl group of Tyr^199^ and O2 of the Gal moiety of UDP-Gal. However, no significant change in the *K_m_* value toward UDP-Gal was observed for the Y199F mutant, indicating that this interaction may not play a critical functional role in nucleotide binding. The location of Tyr^199^ favors a role as contributor to the hydrophobic surrounding of the acceptor substrate binding pocket together with Tyr^194^, Tyr^196^, and Trp^224^ residues when hβ4GalT7 is in its closed conformation (34).

Investigation of the structural role of His^195^ led to the most interesting findings for the design of efficient substrates and inhibitors of hβ4GalT7. We predicted that the nitrogen atom of the peptide backbone of this residue forms a hydrogen bond with the carbonyl group of the coumarin moiety of 4-MUX. This is in full agreement with our mutational study that showed no major effect upon changing the side chain of the histidine residue at this position by alanine, glutamine, or arginine substitution on the hβ4GalT7 activity monitored *in vitro* and *in cellulo*. However, the functional importance of an interaction between the His^195^ backbone and 4-MUX was clearly emphasized by the stronger inhibitory effect of 4-deoxy-Xyl-MU compared with 4-deoxy-Xyl-NP. We also found that 4-MUX was used as a substrate with a better affinity than Xyl-NP (data not shown). Noteworthy, among a series of naphthyl xylosides, Siegbahn *et al.* (29) showed that 6-hydroxy-naphthyl-Xyl was able to prime GAG chains more efficiently than any other unsubstituted derivative in breast cancer cell lines. Altogether, this gives strong evidence that His^195^ through its backbone provides an important structural element for efficient binding of 4-MUX derivatives, and offers a molecular explanation for the superiority of 4-MUX synthesized in this study over naphthyl and benzyl-substituted xylosides previously reported in the literature (25, 30).

We also discovered a unique active site basic residue, *i.e.* Arg^226^. Interestingly, this residue is located between the aromatic-rich ^221^FWGWG^225^ sequence, containing Trp^224^ that interacts with the β-phosphate O*-*atom of the donor substrate, and the ^227^EDDE^230^ sequence containing acidic residues that are involved in Xyl binding and in the transfer reaction (31). Our functional analysis showed that modifying the side chain of Arg^226^ by site-directed mutagenesis did not affect enzyme affinity toward acceptor or donor substrate. This is in line with the computational analysis indicating that the nitrogen atom of the peptide backbone of Arg^226^, but not its side chain, interacts with the O3 atom of the Xyl moiety. Fig. 1, *A* and *B,* clearly shows that this residue, together with Trp^224^, are brought close to the aromatic triad in the closed conformation of hβ4GalT7.

We also investigated the role of Arg^270^, which substitution by a cysteine residue is implicated in the progeroid form of EDS (49). Our previous studies revealed that this genetic mutation dropped *in vitro* hβ4GalT7 activity and impaired GAG chains synthesis in CHO-pgsB618 cells (17). These effects were suggested to be due to a loss of hydrogen bonding between the lateral chain of Arg^270^ and the hydroxyl group of a serine residue from the PG core protein (17). This idea was supported by later molecular modeling indicating that Arg^270^ borders the catalytic site of hβ4GalT7 in the closed conformation (Ref. 34 and this work, see Fig. 1*A*). However, the precise mechanism by which Arg^270^ modulates *in vitro* and *ex vivo* hβ4GalT7 activities remains unclear. Indeed, current crystal structures and molecular models do not point to a specific role of this residue in catalysis or substrate binding, consistent with kinetic data showing that mutations of Arg^270^ in alanine and lysine moderately affect hβ4GalT7 activity and affinity, and the observation that in *Drosophila*, the corresponding position is occupied by a lysine residue. The Arg^270^ residue is located in the flexible loop (261–284) that moves upon donor substrate binding, thus creating the acceptor substrate binding site. This conformational change leads to the closed and catalytically competent conformation of the active site. It thus can be expected that any mutations affecting the loop movement would impair the transfer reaction. However, why the substitution of Arg^270^ by a cysteine residue that causes the progeroid form of EDS patients, produces more deleterious consequences than alanine or lysine mutations requires further investigation of the conformational modifications operating during the catalytic cycle.

Our current and previous functional and computational approaches provide a detailed cartography of the hβ4GalT7 acceptor substrate binding pocket for the rational design of xyloside-based inhibitors (31). We show that the active site organization is governed, on one side, by a series of aromatic amino acids comprising three Tyr residues, *i.e.* Tyr^194^, Tyr^196^, and Tyr^199^, which together with Trp^224^ create a hydrophobic environment and provide stacking interactions essential to the binding of both the xylosyl and aglycone parts of the acceptor substrate. On the opposite side of the site, it involves a network of hydrogen-bonding interactions between three charged amino acids, *i.e.* Asp^228^, Asp^229^, and Arg^226^, and the hydroxyl groups of the Xyl moiety and other active site residues. Until now, most studies aiming to inhibit cellular and extracellular GAG synthesis have been targeted to the synthesis and testing of xyloside derivatives acting as substrates of β4GalT7 thus reducing the glycosylation of endogenous proteoglycan core proteins (19). This approach successfully provided promising pharmacological agents, in particular anti-tumor compounds (45, 50). However, because such molecules behave both as exogenous primers of GAG synthesis and inhibitors of endogenous GAG formation, deciphering their mechanism of action remains challenging. With the perspective of designing xyloside derivatives that selectively act as inhibitors of GAG formation, we opted for C4-modified analogs, whose modification at the C4 position prevents the catalytic transfer, and first synthesized deoxy derivatives. In addition, we took advantage of the information gained from our structural and mutational analyses. We considered two key elements of the aglycone binding, *i.e.* the strategic position of Tyr^194^ that forms stacking interactions with the aglycone part of the acceptor substrate as well as the interaction between His^195^ N-backbone and the carbonyl group of the coumarinyl moiety. In agreement with our prediction, our results clearly show that the 4-deoxy-xyloside appended to 4-MU was superior to the naphthyl-substituted molecule, as indicated by *in vitro* and *in cellulo* studies, supporting the idea that the hydrogen bond between His^195^ and the carbonyl group of the coumaryl group is crucial. Interestingly, Tsuzuki *et al.* (28) found that among triazole xyloside derivatives generated by click chemistry bearing various aromatic and nonaromatic aglycones, the *p*-nitrophenyl analog was the best inhibitor of PG synthesis when screened in endothelial cells. Although detailed docking of these triazole derivatives should be performed, it is tempting to speculate that the formation of a hydrogen bond interaction between the aglycone nitro group and His^195^ enhances the inhibitory potential compared with the other substituted benzyl derivatives. Furthermore, we show here that the 4-deoxy-4-fluorinated 4-MUX was superior to the unsubstituted 4-deoxy analog, indicating that addition of an electronegative atom at this position is an important element in the design of potent inhibitors. The 4-hydroxyl group is involved in two hydrogen bonds with the carboxyl group of Asp^228^ and the 4-hydroxyl group of Gal, respectively. The replacement of the hydrogen donor hydroxyl group by a fluorine atom that is larger than hydrogen and which van der Waals radius and electronegativity are closer to oxygen, at the C4 position, enhanced binding interactions with hβ4GalT7. This corroborated previous studies showing that several fluorinated xylosides act as “good” substrates or inhibitors of GAG synthesis (18, 29, 30). In the same way, fluorinated thrombin inhibitors showed improved factor Xa binding (51). Further docking calculations of fluoro-substituted xylosides are underway to assess the mechanism underlying the improved interactive properties upon fluorine incorporation.

In summary, we developed a powerful approach for the design of xyloside inhibitors that specifically target hβ4GalT7. By integrating structural elements important for the binding of both the Xyl moiety and the hydrophobic aglycone, we synthesized a xyloside-based inhibitor of hβ4GalT7. We generated a compound that both impact *in vitro* galactosyltransferase activity and affect GAG synthesis in cells, opening promising pharmacological applications. This molecule also represents a valuable chemical biology tool to explore the biological effects of GAGs.

## Footnotes

2 The abbreviations used are:

GAG: glycosaminoglycan
EDS: Ehlers-Danlos syndrome
4-MU: 4-methylumbelliferone
NP: naphthyl
PG: proteoglycan
Xyl: xylose
hβ4GalT7: β1,4-galactosyltransferase 7.
